# Inferior mesenteric arteriovenous malformation extending to splenic flexure colonic wall presenting with massive lower gastrointestinal bleeding, a case report

**DOI:** 10.1016/j.ijscr.2023.108322

**Published:** 2023-05-19

**Authors:** Yumna Njoum, Khaled Alshawwa, Bashar Jaber, Omar Abu-Zaydeh

**Affiliations:** a Faculty of Medicine, Al-Quds University, Jerusalem, Palestine; b Department of Surgery, Al-Makassed Charitable Society Hospital, Jerusalem, Palestine

**Keywords:** Inferior mesenteric artery, Arteriovenous malformation, Splenic flexure, Lower gastrointestinal bleeding, Surgical resection, Case report

## Abstract

**Introduction and importance:**

An arteriovenous malformation (AVM) in the inferior mesenteric artery is a rare vascular pathology that accounts for 6 % of the causes of gastrointestinal bleeding. AVMs are typically classified as congenital persisting embryonic vasculature that link both systems and do not develop into arteries or veins [3], but they may also develop later in life. The majority of documented cases are iatrogenic after colon surgery.

**Case presentation:**

Herein we describe a 56-year-old man who presented complaining of fresh blood per rectum with passage of clots not related to defecation with no previous similar episodes, he was found to have extensive inferior mesenteric branches AVM invading the colonic splenic flexure via Computed Tomography (CT) angiography following three non-revealing upper and lower endoscopies which was later managed surgically with left hemicolectomy with primary end to end colo-colic anastomosis.

**Clinical discussion:**

Although AVMs seldom manifest in multiple sites through the gastrointestinal tract, it is more prevalent in the stomach, small intestine, and ascending colon, and very unusual to affect the Inferior mesenteric artery and vein and to extend to the splenic flexure colonic wall.

**Conclusion:**

Even if rare, inferior mesenteric AVMs should be suspected in a patient presenting with GI Bleeding with unrevealing endoscopies, where CT Angiography is to be considered.

## Highlights

• Gastrointestinal vascular lesions, are dilated, weak vessel walls that can rupture into the intestine and cause bleeding, accounting for 6% of gastrointestinal bleeding and more common in the elderly.
• Gastrointestinal vascular malformations are rare to appear in multiple gastrointestinal sites, but it's more common in the stomach, small intestine, and ascending colon. It's unlikely to affect the Inferior mesenteric artery and vein or extend to the splenic flexure colonic wall.
• Possible differential diagnoses include cancerous tumors, inflammatory bowel conditions, coagulopathy, and anorectal infections/illnesses.
• Diagnosis is best achieved by selective mesenteric CT Angiography. However, the final diagnosis relies on gross and histologic demonstration of vascular malformations in the excised specimen.
• Vascular lesions of the colon can be colonoscopically ligated, endovascularly embolized, or surgically removed, which is case dependent option.

## 1 Introduction

Arteriovenous malformations (AVMs) are gastrointestinal vascular lesions and account for 6 % of the causes of gastrointestinal bleeding with Inferior mesenteric (IM) vessels being least commonly involved. [1] IM AVMs invading the colonic wall at the level of the splenic flexure as in our case is considered a very rare entity of IM AVMs and a rare etiology of rectorrhagia which is considered as a nonspecific complaint, differential diagnosis includes cancerous tumors, inflammatory bowel conditions, coagulopathy, and anorectal pathologies. Colonoscopies are the first used diagnostic method for finding colonic AVMs since colon cancer is the primary differential diagnosis to be ruled out. [1] However its sensitivity is highly variable with multiple limitations in diagnosing gastrointestinal bleeding secondary to AVMs, Hence, CT angiography has become more informative which shows dilated vessels with early-filling veins, and an enlarged supplying artery. [1] Management of this condition is case-dependent and requires risk-benefit analysis as discussed in our case relies on the extent and size of the malformation, patient comorbidities, possible complications, and challenges for each management option. It can be colonoscopically ligated, endovascularly embolized, or surgically removed.

After reviewing the literature, there were a very limited number of reported cases of IM AVMs with multiple presentations, including ischemic colitis, portal hypertension, liver congestion, incidental discovery, and very limited cases of massive lower gastrointestinal bleeding. Our case presented with IM AVM extending up to the splenic flexure colonic wall which to our best knowledge, this extent was not reported before, further complicated with massive bleeding diagnosed after multiple trials of upper and lower endoscopies and capsule endoscopy and managed with surgical resection after multidisciplinary team decision after discussing management options.

## 2 Case presentation

**Figs. 1, 2, 3:**
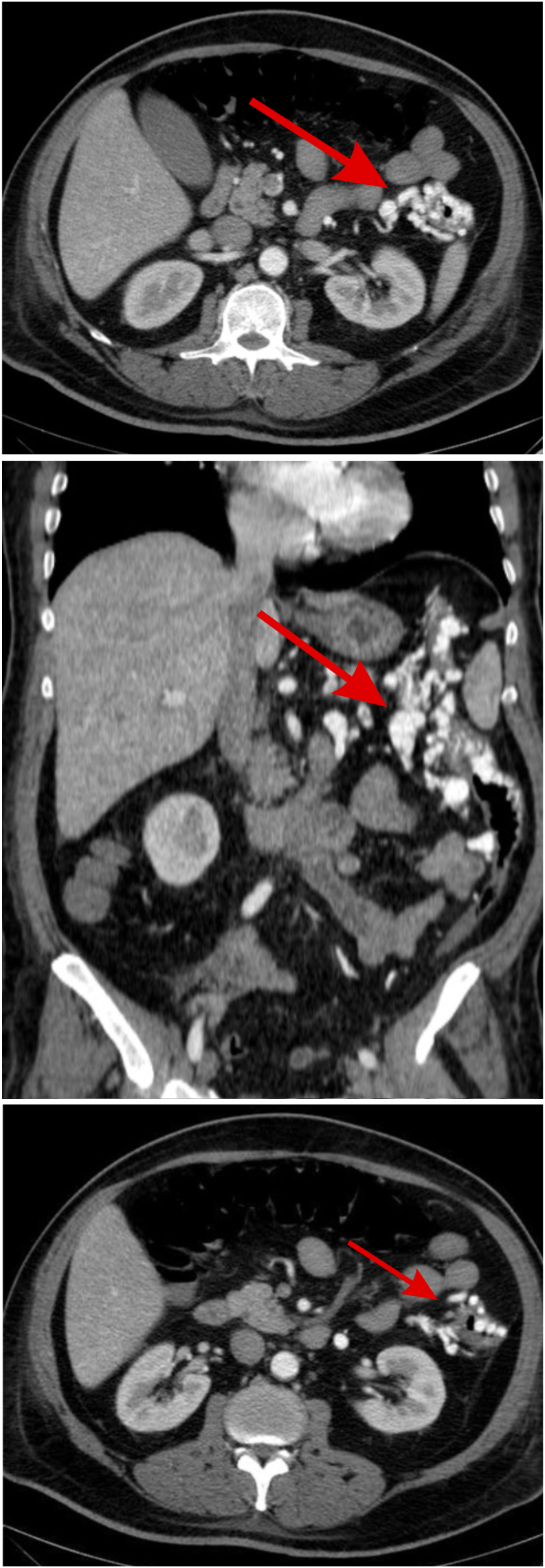
Axial and coronal CT cuts showing extensive serpiginous enhancing tubular structures affecting left colon wall, noted mainly in the arterial phase. Findings are in-keeping with left colon angiodysplasia, associated with enlarged IMA and its feeding arteries and draining veins. The disease extends to the splenic flexure. The sigmoid is free of disease.

A 56 year-old male with diabetes, presented with a 10 days history of multiple episodes of spontaneous painless lower gastrointestinal bleeding requiring massive blood transfusion and symptoms of anemia, Bleeding contained fresh blood and blood clots. After stabilization with >20 Packed red blood cells units, Hemoglobin level ranged from 6.0 to 9.0 g/dL. The patient underwent multiple upper and lower gastrointestinal endoscopies and video capsule endoscopy, without definitive localization of the source of bleeding as there were multiple angiodysplastic lesions in stomach, duodenum, and colon with no obvious active bleeding. He underwent abdomen CT-Angio which revealed extensive serpiginous enhancing tubular structures in-keeping with left colon angiodysplasia extending to the splenic flexure associated with enlarged inferior mesenteric trunk and its feeding arteries and draining veins (Figs. 1, 2, 3).

**Fig. 4:**
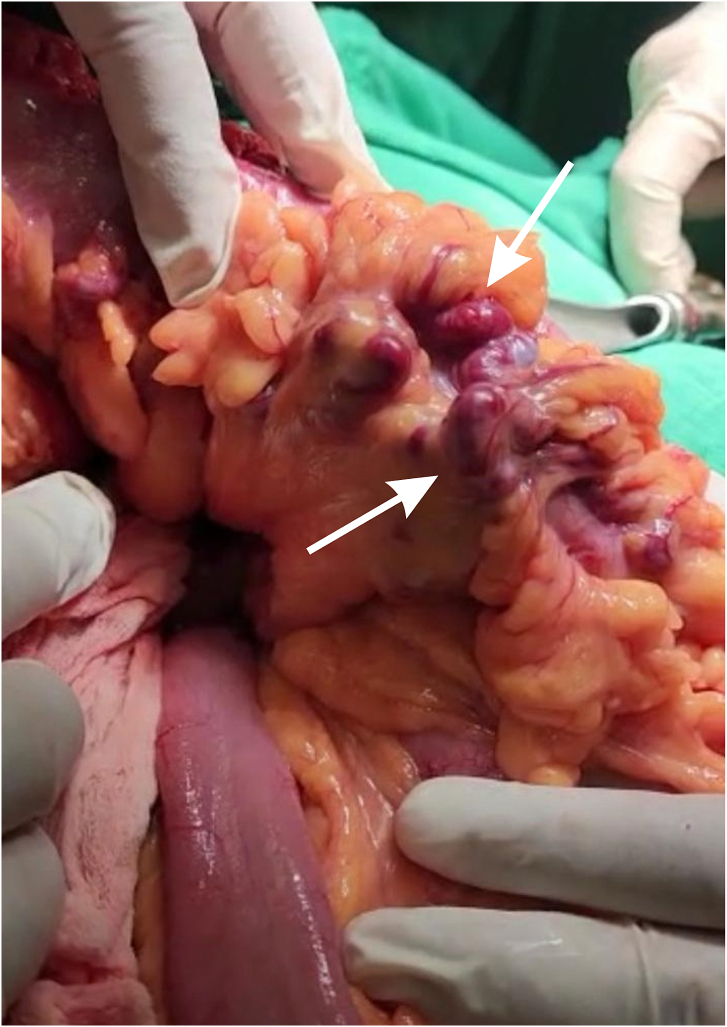
Intraoperative finding of tortuous dilated arteriovenous malformation involving mainly the wall of left colon and splenic flexure (arrow).

There was no possibility of embolization due to inability to identify a single feeding vessel as there was an extensive large AVM. So he underwent exploratory laparotomy by a colorectal surgeon, there was grossly severely dilated veins of splenic flexure down to sigmoid colon, draining into IMV, with extraluminal extensive AVM of left colon, feeding vessels were mainly from ascending branch of left and middle colic arteries involving left colon mainly splenic flexure (Fig. 4), they were isolated and ligated by two Hemolocks, left hemicolectomy with primary end to end colo-colic anastomosis was performed, one Jackson-Pratt drain was inserted. Patient was transferred to the Intensive Care Unit for post-operative observation, course went without complications, required no blood transfusion and no episodes of active bleeding occurred. He was discharged home 4 days later. Histopathologic examination of the resected segment showed irregular blood-filled spaces with focal spread to the mucosa and submucosa, Features are consistent with intestinal vascular malformation.

Upon follow-up one month post operatively, he reported being well, with no recent post-management complaints, he denied abdominal pain, diarrhea, constipation, hematochezia, fatigue, exertional dyspnea, lightheadedness or symptoms of anemia.

Upon examination, he was vitally stable, well looking, with a well healed laparotomy scar. Abdomen was soft lax with no tenderness to palpation. Rectal examination showed no dark tarry stool or palpable hemorrhoids.

He also did complete blood count and showed hemoglobin levels of 13 g/dL (discharged with hemoglobin of 10.5 g/dL).

## 3 Discussion

Gastrointestinal AVM is often solitary and has no defined location in the GI tract nor confined to old age groups, in which the arteries and veins are directly joined with no intervening capillary beds. Inferior mesenteric AVMs presentation is highly variable, from incidental finding through screening colonoscopies or during imaging for different purpose, ischemic colitis after demand supply mismatch due to steal phenomena causing anginal abdominal pain, to portal hypertension and liver congestion, and even life-threatening lower GI bleeding. [2] Misdiagnosis is the major problem in these patients. Patients with chronic lower gastrointestinal bleeding should be evaluated for arteriovenous malformations. With colon cancer the main differential diagnosis because of the common clinical presentation and age group, where all intentions are towards exclusion of colon cancer. Colonoscopies have become the main diagnostic tool to discover colonic AVMs as a first line diagnostic option of lower GI Bleeding. Imaging still plays a significant role in the diagnosis of GI bleeding, though. It is crucial to fully comprehend the role of interventional radiology and endovascular treatment of GI hemorrhage, due to the wide variety of causative etiologies, high possibility of obscure bleeding, and long length of the GI tract, which all lie as obstacles in the way of endoscopy, and the restriction of assessment to the mucosal component of the AVM when succeeding to visualize the lesion due to the inappropriate preparation of bowel either because of emergency presentation of massive hemorrhage or due to excessive blood clots in the colon after bleeding. as what case has faced 3 times failed upper and lower endoscopies and failed once video capsule endoscopy. [1] Leaving diagnosis best achieved by selective mesenteric CT Angiography as in our case. However, the final diagnosis relies on gross and histologic demonstration of vascular malformations in the excised specimen. [3] The advantages of interventional radiology and endoscopic therapy include the ability to re-procedure, being minimally invasive, cost-effective, and suitable for high-risk patients. And were described to be safer in management of solid organs malformations, but these techniques have possible recurrence so surgical excision is frequently seen as the most effective management. Endovascular embolization is not recommended in large AVMs >8 mm diameter, hollow viscus organs, or with high-flow AVMs due to the risk of nontarget distal embolization, thrombosis, and ischemia. In these cases, the treatment is only surgical. [2] Literature showed right hemicolectomy as the most common surgical procedure performed in adults with colonic AVMs indicating higher prevalence than left sided colonic AVMs. [4] Patients should be followed up for early and late surgical complications of colectomy including leak, infections, ileus, fistula formation and obstruction via physical examination and proper diagnostic imaging. Also, he should be assessed for vascular integrity and symptoms of end organ hypoperfusion after resection, or recurrence of the vascular malformation.

As well as recurrence of anemia and lower gastrointestinal bleeding via vital signs monitoring, rectal examination, stool for occult blood test and hemoglobin levels.

## 4 Conclusion

Lower gastrointestinal bleeding is an alarming sign and requires prompt evaluation, CT angiography provides high sensitivity and specificity for diagnosing AVMs of the intestine and should be considered early in diagnosis.

AVM should be suspected in patients presenting with painless massive lower gastrointestinal bleeding even with no previous history of intestinal surgeries for better diagnosis and treatment of this challenging condition and improve their ability to diagnose and treat it promptly and effectively.

## 5 Methods

Work has been reported in line with the SCARE criteria [5].

## Declaration of competing interest

There is no conflict of interest.
